# Stochastic Magnetization Switching in Rapidly Solidified (Co_0.94_Fe_0.06_)_72.5_Si_12.5_B_15_ Amorphous Submicronic Wires

**DOI:** 10.3390/ma15030896

**Published:** 2022-01-25

**Authors:** Sorin Corodeanu, Costică Hlenschi, Cristian Rotărescu, Horia Chiriac, Nicoleta Lupu, Tibor-Adrian Óvári

**Affiliations:** National Institute of R&D for Technical Physics, 47 Mangeron Boulevard, 700050 Iași, Romania; scorodeanu@phys-iasi.ro (S.C.); chlenschi@phys-iasi.ro (C.H.); crotarescu@phys-iasi.ro (C.R.); hchiriac@phys-iasi.ro (H.C.)

**Keywords:** metallic glasses, magnetic wires, magnetization switching, magnetic domain walls

## Abstract

Submicrometric magnetic amorphous wires are good candidates for future development of miniaturized sensors and magnetic logic applications. Here we report the results of an in-depth investigation of magnetization switching in rapidly solidified nearly zero magnetostrictive (Co_0.94_Fe_0.06_)_72.5_Si_12.5_B_15_ amorphous samples with diameters of the actual magnetic wires between 300 and 450 nm. All samples were found to be magnetically bistable, displaying characteristic rectangular hysteresis loops. This shows that magnetization reversal occurs through the depinning and subsequent propagation of a magnetic domain wall, whose velocity depends on the applied field and on the sample dimensions. The results of this study reveal stochastic nonlinear dependencies of both the magnetic switching field and the domain wall velocity on the sample diameter. The analysis of the potential causes, which include nonlinear residual stresses, fluctuations in wire dimensions (metal and glass), and competing magnetic anisotropies of different origins, show that a combination of all three factors could lead to the observed stochastic behavior. Calculated values of the switching field, which consider only changes in the wire dimensions, indicate that such influence alone cannot account for the strong nonlinearities. The results are important for the applications of such ultrathin cylindrical magnetic amorphous wires.

## 1. Introduction

Rapid solidification has been used to prepare cylindrical wire-shaped amorphous metals since the 1970s [1]. Using various melt spinning techniques, e.g., glass-coated melt spinning, in-rotating-water spinning, amorphous metallic wires with diameters between a few micrometers and over 120 μm in diameter have been widely produced and investigated [2]. Among these, typical families of highly magnetostrictive, e.g., Fe_77.5_Si_7.5_B_15_, nearly zero magnetostrictive, e.g., (Co_0.94_Fe_0.06_)_72.5_Si_12.5_B_15_, and negative magnetostrictive, such as Co_80_Si_10_B_10_, magnetic wires and microwires have been prepared and studied [3]. Much more recently, rapid solidification has been employed to prepare and investigate submicronic amorphous wires [4,5]. These materials offer important benefits in terms of miniaturization, as well as of new application opportunities for much needed state-of-the-art purposes, such as micro and nano-sensing devices [6], medical applications [7,8,9], information technology engineering applications in magnetic logic devices [10], flexible electronics, various functional devices and smart structures [11]. They could also be employed as key elements in miniaturized tunable metamaterials, adjustable microwave materials, as well as self-sensing micro and nanomaterials [12].

These ultrathin cylindrical amorphous magnetic wires exhibit many advantages in comparison with other types of magnetic materials having transverse dimensions within the same range (hundreds of nm), e.g., electrodeposited arrays of nanowires [13] or planar and cylindrical wires made by various lithography techniques [14]. These advantages refer to a more suitable overall shape for the fast and ultrafast propagation of magnetic domain walls (vs. the planar samples), much longer wire length that allows the approach and development of different types of new applications (vs. the electrodeposited and lithographically made samples), absence of array neighbors that create unwanted dipolar bias field (vs. electrodeposited arrays of nanowires), and the inherent advantage of the amorphous phase, i.e., the absence of the magnetocrystalline anisotropy (vs. lithographically prepared wires).

The aim of this work is to perform an in-depth investigation of magnetization switching in rapidly solidified nearly zero magnetostrictive (Co_0.94_Fe_0.06_)_72.5_Si_12.5_B_15_ amorphous wires with diameters between 300 and 450 nm, prepared by an improved variant of the glass-coated melt spinning method. The study, initially aimed towards the accurate control over the magnetization switching process and its underlying mechanism—the depinning and propagation of the 180° magnetic domain wall—has revealed an unexpected stochastic behavior of both the actual magnetization switching and the velocity of the associated domain wall. The results are important as concerns the basic magnetic behavior of nearly zero magnetostrictive amorphous submicronic wires with diameters below 500 nm, the actual limits of glass-coated melt spinning in the preparation of wires with such small diameters having predictable magnetic properties, and their future applications.

## 2. Materials and Methods

**Figure 1:**
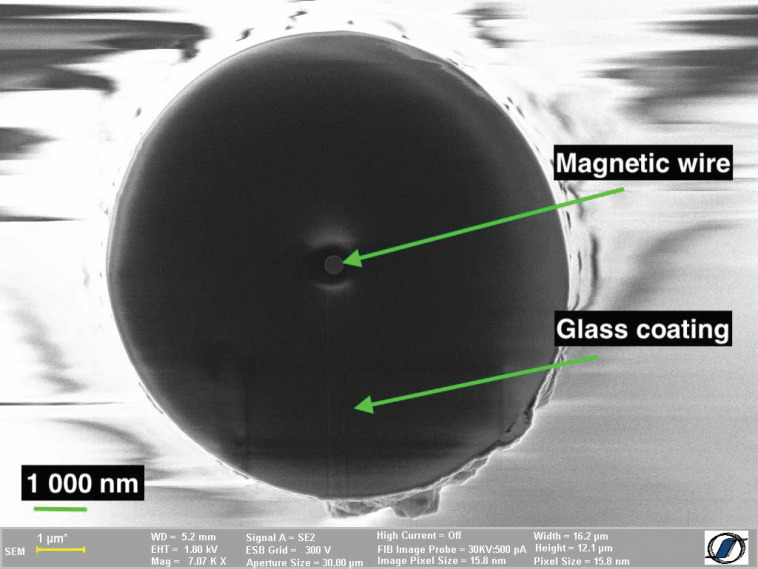
SEM image of a (Co_0.94_Fe_0.06_)_72.5_Si_12.5_B_15_ amorphous glass-coated submicronic wire sample. The actual metallic wire is the bright dot-like object in the middle, the rest being glass.

The (Co_0.94_Fe_0.06_)_72.5_Si_12.5_B_15_ amorphous submicronic wires employed in this investigation were prepared using the improved variant of the glass-coated melt spinning method developed at the National Institute of Research and Development for Technical Physics in Iași, Romania [15]. A typical scanning electron microscopy (SEM) image of such a sample is shown in Figure 1. The technique is based on several key enhancements over the classical method that allow the fabrication of such ultrathin wires. The main ones refer to the actual drawing speed of the softened glass capillary in which the molten alloy is flowing, to the suppression of the vibrations in the entire wire making equipment, and to the adjustment of the other process parameters to the requirements of such small diameters. Using this method, we prepared in a single-step process amorphous glass-coated submicronic wires with the diameters of the actual metallic wires from 300 to 450 nm, and the total diameter (metallic wire + glass coating) between 8.4 and 11.4 μm. The ratio between the radius of the metallic wire and the glass coating thickness varies between 0.038 and 0.047. Although the samples prepared through this rapid solidification method are very long—tens and hundreds of cm—for the actual magnetic measurements we have employed much shorter pieces, i.e., 4 cm, by cutting them from the long as-cast wires. The integrity of the samples was visually checked using a Carl Zeiss AXIO IMAGER A1m optical microscope (Carl Zeiss MicroImaging GmbH, Göttingen, Germany).

Magnetostriction measurements were performed by the small angle magnetization rotation (SAMR) method, previously adapted for microwires [16], and further enhanced for submicronic samples.

The magnetic measurements included magnetic hysteresis loop measurements and measurements of the velocity of the magnetic domain wall associated with the magnetization switching process, and which propagates along the wire axis.

Axial hysteresis loops have been measured using an inductive technique specifically devised for such thin cylindrical wires that exhibit a magnetically bistable behavior [17], i.e., in which the axial magnetization is reversed in a single step once the axially applied magnetic field reaches the threshold value called switching field, H*. The method is based on a digital integration technique, which allows one to measure noise-free hysteresis loops [18]. It uses significant signal amplification in order to reach a sufficiently large value of the induced voltage and a large enough signal-to-noise ratio.

The magnetic domain wall velocity measurements have been performed by means of a Sixtus–Tonks-based method [19], adapted for the specific small cross sections of the investigated magnetic submicronic amorphous wires [20]. There were several improvements made in order to avoid errors generated by the potential nucleation of additional domain walls, such as the use of multiple pick-up coils (more specifically, two compensated sets of four pick-up coils), as well as other enhancements in terms of sensitivity, signal amplification, and processing, to compensate for the initially low signal-to-noise ratio. Moreover, from a practical point of view, the wall velocity measuring system has gained the capability of reading the direction of the domain wall displacement by means of opposite direction windings in the pick-up coils. The maximum value of the axially applied magnetic field in our domain wall propagation experiments was *H_max_* = 10,000 A/m.

## 3. Results

All the investigated samples have been found to display rectangular axial hysteresis loops, a clear indication of the magnetically bistable behavior. Although the analyzed samples have a nearly zero saturation magnetostriction constant, i.e., *λ_S_* = −1 × 10^−7^, which generally translates into a reduced magnetoelastic contribution to the overall magnetic anisotropy, they exhibit this magnetic bistability, which is a completely different behavior as compared to the magnetic behavior of amorphous glass-coated microwires with the same composition [21].

**Figure 2:**
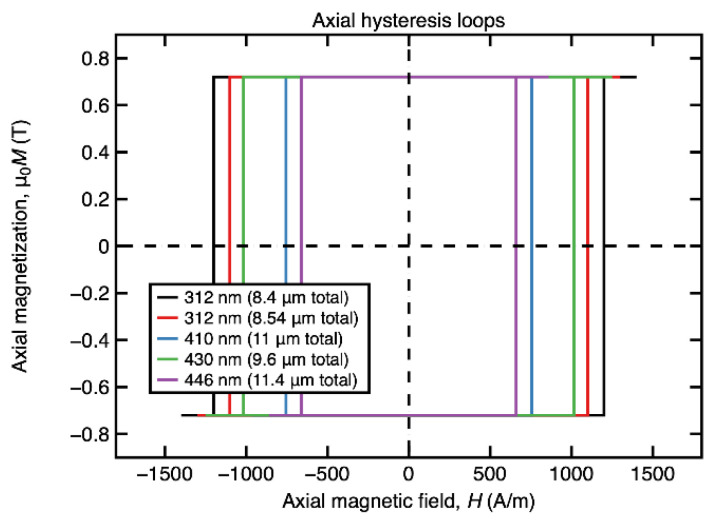
Axial hysteresis loops of (Co_0.94_Fe_0.06_)_72.5_Si_12.5_B_15_ amorphous submicronic wire samples with nearly zero magnetostriction.

Figure 2 illustrates the axial magnetic hysteresis loops of several (Co_0.94_Fe_0.06_)_72.5_Si_12.5_B_15_ submicronic amorphous wire samples. One observes that for such small diameters, the switching field has relatively low values, which makes them magnetically soft for this class of materials. The mechanism of axial magnetization reversal consists in the depinning and subsequent propagation of a preexisting 180° magnetic domain wall. This domain wall preexists towards a wire end due to the demagnetizing effect. When the applied field *H* reaches or exceeds the value of the switching field H* (*H* ≥ H*), the depinning of the wall occurs and it immediately propagates towards the other end of the wire, resulting in the reversal of the magnetization.

**Figure 3:**
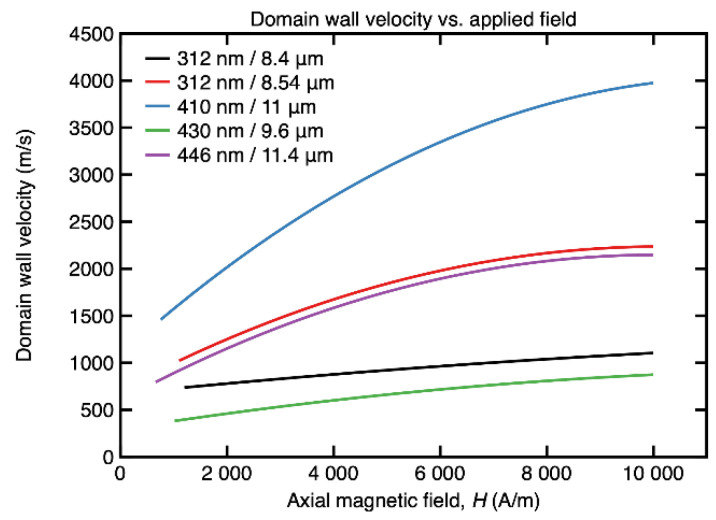
Field dependence of the magnetic domain wall velocity for (Co_0.94_Fe_0.06_)_72.5_Si_12.5_B_15_ amorphous submicronic wire samples with nearly zero magnetostriction.

The propagation velocity of this 180° magnetic domain wall was measured for values of the axial magnetic field between the switching field in the case of each sample and the maximum applied field, which is 10,000 A/m in this study (H* ≤ *H* ≤ *H_max_*). In all cases, the domain wall velocity increases monotonically with the applied field, which is a typical behavior for all amorphous ferromagnetic wires [22]. The curves that illustrate the field dependence of the magnetic domain wall velocity for the investigated (Co_0.94_Fe_0.06_)_72.5_Si_12.5_B_15_ submicronic amorphous wire samples are shown in Figure 3.

One observes that, for the sample having 410 nm in diameter, the domain wall velocity increases from about 1500 m/s up to a maximum of nearly 4000 m/s at *H* = *H_max_*, which is a rather large value, more than double the value at *H* = H*, of interest for applications based on fast magnetic domain walls, such as magnetic logic or even magnetic delivery of functionalized nanoparticles.

**Figure 4:**
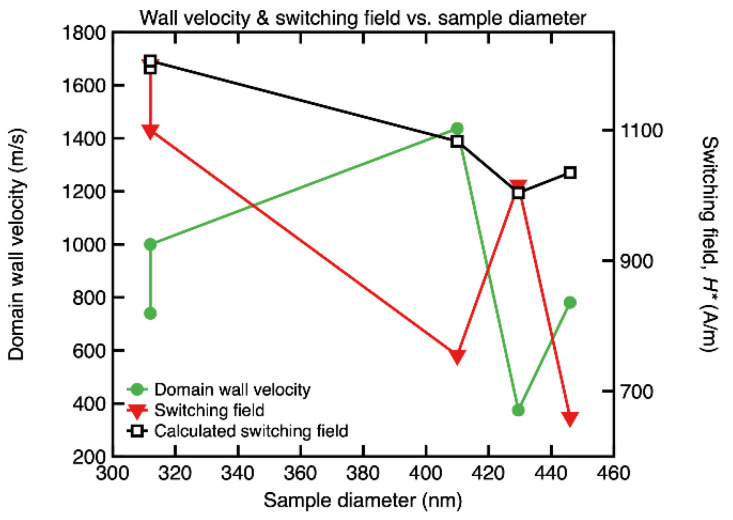
Magnetic domain wall velocity at *H* = H* and switching field vs. sample diameter for (Co_0.94_Fe_0.06_)_72.5_Si_12.5_B_15_ amorphous submicronic wires.

From the hysteresis data and from the results of the domain wall velocity measurements for all the investigated samples, we have extracted the dependence of the switching field and of the domain wall velocity at various values of the applied field, as a function of the diameter of the submicronic amorphous wires, both being illustrated in Figure 4. One can observe that both quantities exhibit a strongly nonlinear dependence on sample diameter. Furthermore, the slopes of the two curves are always opposite to one another. These nonlinear variations are the most remarkable and, at the same time, the most puzzling results of this investigation.

The nonlinear shape of the domain wall velocity vs. sample diameter curve that corresponds to *H* = H* is maintained even at larger values of the axially applied field, up to *H* = *H_max_* (10,000 A/m).

## 4. Discussion

Submicronic amorphous wires with nearly zero magnetostriction are intriguing in the first place due to their different magnetic behavior in comparison with their larger counterparts, the microwires with similar magnetostriction, with the submicronic ones being bistable and the micrometric ones not. Previous studies have established the importance of shape anisotropy, of magnetostatic origin, in the specific magnetic behavior of nearly zero magnetostrictive amorphous submicronic wires [23]. Nevertheless, a more thorough investigation of the relation between sample diameters and the size-induced magnetic bistability (it occurs only in the submicronic samples) in nearly zero magnetostrictive submicronic amorphous wires, is expected to be important both for better understanding their intrinsic properties, as well as for their potential applications. Hence, the most representative physical quantities that are directly linked to the bistable magnetization reversal process, i.e., the switching field and the associated magnetic domain wall velocity, have been used for such an investigation. Both quantities display a nonlinear dependence on the sample diameter, in such a way that it can be clearly stated that neither the switching field, nor the domain wall velocity can be accurately predicted for a certain wire diameter. In the 300 nm to 450 nm range of diameters, for the specific composition considered—(Co_0.94_Fe_0.06_)_72.5_Si_12.5_B_15_—that is one of the most representative compositions for nearly zero magnetostrictive amorphous alloys, both the switching field and the domain wall velocity seem to have a mostly random dependence on wire diameter.

The nonlinear dependencies can originate in the following three factors, most likely due to their combination:

1. the nonlinear distribution of the frozen-in internal stresses generated by the rapid solidification preparation process;
2. the fluctuations in the metallic wire diameter, in the glass coating thickness, or in both—this also includes the cases in which the metallic wire might not be accurately centered within the glass. These fluctuations are on the wire length and there is a clear connection to the first factor above (internal stresses); and
3. the (Co_0.94_Fe_0.06_)_72.5_Si_12.5_B_15_ amorphous submicronic wires have competing anisotropies of the same order—the shape anisotropy (magnetostatic) and the magnetoelastic anisotropy—which still has an important contribution despite the small magnetostriction, due to the significant internal stresses generated by the rapid solidification preparation process.

A typical nonlinear internal stress distribution is given in [23]. Maximum values are of several GPa, and they change from tensile (positive) in the inner part of the wire to compressive (negative) in the near-surface region. This obviously affects the depinning field, the local distributions of the magnetization in the inner part and in the surface region, which can both affect the value of the switching field and the domain wall velocity (the latter through the stray fields created by the unfavorable distributions of magnetization).

Fluctuations in wire diameter and glass coating thickness have been previously shown to affect the values of the magnetic switching field in the larger amorphous glass-coated microwires, in which fluctuations of up to 10% have been reported [24]. We have also employed the equation provided for microwires in [24] to calculate the switching field values that would result for the dimensions of the investigated submicronic amorphous wires (metal, glass, their ratio). The results are plotted on Figure 4, together with the experimental values. One observes that, even though the values of the switching field are of the same order, the predicted variation, which originates in changes of both metallic wire diameter and glass coating thickness (as both are parameters in the equation, alongside their ratio), is clearly less pronounced than the actual experimentally observed variation. This supports our hypothesis that a combination of the three factors mentioned above is the most probable cause of the random behavior of the switching field and domain wall velocity, rather than just one of them, e.g., dimensions and their fluctuations.

The most complicated effect is most likely caused by the competition between the magnetoelastic and magnetostatic contributions to the overall magnetic anisotropy. This situation is unique for these low magnetostrictive submicronic wires, because in the highly magnetostrictive ones, the magnetoelastic contribution is one to two orders larger, whilst in the microwires with nearly zero magnetostriction, the shape anisotropy is not that significant. This again strengthens the idea that all three factors are expected to play key roles in causing the nonlinear variations shown in Figure 4.

Future research will focus on performing a similar study on highly magnetostrictive submicronic amorphous wires, as well as on the actual visualization of the domain walls in such thin samples. Depending on the results of the future investigations, one might be able to shed further light on the influence of each of the above-mentioned factors, given that the last one is essentially missing in the wires with large magnetostriction.

## 5. Conclusions

The investigation of the magnetization reversal process in amorphous glass-coated submicronic wires with nearly zero magnetostriction revealed stochastic type strongly nonlinear variations of the magnetic switching field and domain wall velocity with the sample diameter. The potential causes of this behavior were identified as: (i) the nonlinear distribution of the residual internal stresses; (ii) the fluctuations in the wires’ transverse dimensions (actual metallic wire, glass thickness, their ratio); and (iii) the competing shape and magnetoelastic anisotropies in low magnetostrictive samples. Switching field calculations based on sample dimensions confirm that the interplay of these three factors is expected to be responsible for the observed stochastic magnetization switching. However, further work is still needed in order to completely elucidate the precise contribution of each factor.

The results contribute to the overall understanding of the basics of magnetization reversal in such thin submicronic amorphous wires, and are part of the efforts to accurately control and tailor their magnetic characteristics for future applications, mainly in miniaturized sensors and domain-wall-logic-based devices.

The results also indicate a lower transverse dimensional limit of the specific rapid solidification technique, i.e., of the glass-coated melt spinning method. Thus, in the case of amorphous alloys with low magnetostriction, at such small transverse dimensions, the magnetic properties of the as-cast wire samples cannot be controlled with high accuracy.

## Data Availability

The data presented in this study are available on request from the corresponding author.
